# When the cure turns toxic: a case report on toxic alkaloids identified by public mass spectral databases

**DOI:** 10.3389/fmed.2025.1681334

**Published:** 2025-10-20

**Authors:** Yuyan Lei, Qin Yi, Junli Lu, Yanghao Sheng, Ying Xue

**Affiliations:** ^1^Phase I Clinical Trial Laboratory, The Second Nanning People’s Hospital, Nanning, Guangxi, China; ^2^Department of Pharmacology, School of Pharmaceutical Sciences, Central South University, Changsha, Hunan, China; ^3^Department of Pharmacy, Zhuzhou Central Hospital, Zhuzhou, Hunan, China; ^4^Department of Pharmacy, Xiangya Hospital, Central South University, Changsha, China; ^5^Institute for Rational and Safe Medication Practices, National Clinical Research Center for Geriatric Disorders, Xiangya Hospital, Central South University, Changsha, China

**Keywords:** aconitine poisoning, herbal medical wine, mass spectral databases, Global Natural Product Social (GNPS), traditional Chinese medicine (TCM)

## Abstract

Aconitine is a highly toxic diterpenoid alkaloid, produced by root of *Aconitum brachypodum* Diels. (*A. brachypodum*), also known as “Xue-Shang-Yi-Zhi-Hao,” that is still used in Chinese herbal medicines. Aconitine poisoning remains common in China and other parts of Asia. *Cistanche deserticola* Y. C. Ma (CD), a drug-food homologue plant, bears a resemblance to *A. brachypodum*, thus posing a risk of accidental ingestion. Here we present a case report of aconitine poisoning resulting from accidental ingestion. A 54-year-old male presented to the emergency department with toxic symptoms after ingesting homemade herbal medicinal wine. Toxicological analysis was performed, the herbal medicinal wine sample retained from the patient was analyzed using ultrahigh performance liquid chromatography quadrupole-time-of-flight mass spectrometer system. By utilizing the spectral libraries within Global Natural Product Social (GNPS), we identified several aconitum alkaloids—including indaconitine, yunaconitine, talatisamine, and chasmanine—from the herbal medicinal wine sample. This is the first case report of aconitum poisoning where a large-scale public mass spectral databases was used for the rapid screening of toxic substances. The method applied in this study provides a novel approach for the screening of cases involving unexplained poisoning.

## 1 Introduction

*Aconitum brachypodum* Diels. (*A. brachypodum*), a member of the Ranunculaceae, is chiefly distributed in the northwestern areas of Yunan and the southwestern part of Sichuan in China. The dried tuberous roots of *A. brachypodum*, referred to as Xue-Shang-Yi-Zhi-Hao, has been used as an essential drug in Traditional Chinese Medicine for more than 2,000 years (1, 2). Although *A. brachypodum* derivative compounds such as aconitine and related alkaloids which have been used to treat arthritis, rheumatism and different kinds of pain (3, 4). However, *A. brachypodum* is a toxic herb and can cause fatal cardiac poisoning, there is no antidote in case of overdoses or intoxication resulting from misuse (5). Because of faulty processing, overdosing, and drinking herbal medicinal wine made with alkaline aconitine herbs, aconitine poisoning remains common in China and other parts of Asia (6–8). During 1993–2005, approximately 5,000 cases of aconite poisoning incidents were reported in China, Germany, Japan and other countries; most cases of fatal poisoning occurred in China (9).

*Cistanche deserticola* Y. C. Ma (CD) is a drug-food homologue plant that grows in arid or semi-arid regions. It is known as “desert ginseng” and has been historically used as a significant tonic in conventional Oriental medicine. According to ancient Chinese medical books, it can be used to tonify kidneys, treat impotence and female infertility, relieve constipation, strengthen the bone marrow, and enhance gastrointestinal function (10). The specimen of CD is flat cylindrical, with a surface that is brownish-brown or gray-brown, similar to *A. brachypodum*. Therefore, it is difficult for untrained non-professionals to distinguish between the two Chinese medicine, which may lead to the misidentification of *A. brachypodum* as CD, resulting in accidental ingestion and subsequent poisoning.

Various toxic symptoms, such as paresthesia, anesthesia, and weakness, are observed within a few minutes of *A. brachypodum* ingestion and are followed by gastrointestinal symptoms, such as nausea, vomiting, diarrhea, and cardiac problems, the most common of which is ventricular arrhythmia (11). Although the toxic effects of aconitine alkaloids have been recognized for many years, identifying the specific toxic substance remains crucial in the acute rescue and treatment of affected patients.

In recent years, the application of large-scale public mass spectrometry databases, such as the Global Natural Products Social (GNPS), has become increasingly widespread in the identification of small molecules in complex biological samples (12–14). As a community-managed open platform, GNPS has emerged as a crucial resource for the identification of natural products and toxins. However, its use in clinical acute poisoning scenarios remains in its nascent stages. Notably, there has yet to be any published reports on its real-time toxicological analysis in human acute poisoning events. Against this backdrop, the present case represents the first application of GNPS for the rapid structural elucidation of aconitine alkaloids in a clinical poisoning context, demonstrating the potential of this technology as a novel tool in emergency toxicology.

We herein report a clinical case that demonstrates this novel application: a 54-year-old man drank 75 mL of herbal medicinal wine to tonify kidneys. A few minutes later, he developed abdominal discomfort, and after 1 h, he began to experience profuse sweating, accompanied by vomiting and fatigue, and was subsequently transferred to the emergency room. To confirm the substance responsible for the poisoning, a chemical component analysis was conducted using a LC-QTOF-MS/MS system and samples of herbal medicinal wine were analyzed.

## 2 Methods

### 2.1 Ethics statement

This case report is based on a single case from routine clinical practice and did not involve any interventions beyond standard clinical care. All procedures followed were in accordance with the ethical standards of the institutional and national research committee and with the Helsinki Declaration. Patient privacy was strictly protected, and all identifiable information has been anonymized. In accordance with the policy of the Medical Ethics Committee of Xiangya Hospital, this type of case report is exempt from ethics review, and an official waiver was obtained. Written informed consent was obtained from the patient for the publication of this case report.

### 2.2 Chemicals and regents

HPLC grade acetonitrile was purchased from Merck Inc. (Germany) and HPLC grade formic acid and ammonium formate was purchased from Sigma Inc. (United States). Purified water was supplied by China Resources C’estbon Beverage (China) Co., Ltd.

### 2.3 Sample preparation

The herbal medicinal wine sample was stored in a sealed amber glass container at 4 °C from the time of collection until analysis. A 200 μL homemade herbal medicinal wine sample was dilution with 1 mL of methanol, and sonicated for 30 min. The sample mixture was centrifuged at 5,000 rpm for 15 min, then filtered through a 0.22 μm microfiltration membrane and 5 μL aliquot was injected into ultrahigh performance liquid chromatography (UHPLC) quadrupole-time-of-flight (Q-TOF) mass spectrometer system. The entire process from sample preparation to LC-QTOF-MS analysis was completed within 24 h to minimize potential degradation.

### 2.4 LC-QTOF-MS/MS

The prepared samples were analyzed using a 1290 Infinity UHPLC system coupled to a 6545B Q-TOF mass spectrometer (Agilent Technologies, Palo, CA, United States) equipped with a dual Agilent Jet Stream electrospray ionization source (AJS-ESI). Data acquisition and processing were carried out using the MassHunter workstation software.

Chromatographic separation was performed on a Waters HSS T3 column (100 mm * 2.1 mm, 1.8 μm). The flow rate of the mobile phase was 0.2 mL/min, the column temperature was maintained at 40 °C and the injection volume was 5 μL. The mobile phase was composed of solvent A (0.1% formic acid with 5 mM ammonium formate in water) and solvent B (0.1% formic acid in acetonitrile) and the gradient program was as follows: 10–95% B for 0.0–15.0 min, 95% B for 15.0–20.0 min, and final re-equilibration for 8 min to the initial condition before each injection.

The parameters of the 6545B Q-TOF mass spectrometer, operating in positive mode, were the following: the capillary voltage, nebulizing gas and fragmentation voltage were set to 3,500 V, 35 psi and 160 V, respectively. The drying gas temperature and the drying gas flow were set to 350 °C and 11 L/min, respectively. The sheath gas temperature and the sheath gas flow were set to 350 °C and 10 L/min, respectively. Parameters for the auto-MS/MS mode were as follows: *m*/*z* = 50–1,000, scan rate 3 spectra/s. The collision energy was set 20 eV and 40 eV. Data were acquired in centroid mode at the extended dynamic range mode (2 GHz) over a mass range of 0–1,000 *m*/*z*. The mass calibration of the TOF system was continuously controlled by measuring the protonated reference ions of purine (*m*/*z* 121.050873) and HP-0921 (*m*/*z* 922.009798).

### 2.5 GNPS Library spectral matching

All MS2 spectra were triggered at the apex of the chromatographic peak to ensure high spectral quality and comparability, thereby avoiding potential fragment ion ratio biases associated with peak fronting or tailing. The raw MS/MS data were converted to mzML format using MSConvert (ProteoWizard). The resulting files were then subjected to spectral library searching against the GNPS public MS/MS spectral databases^1^ (15). Spectral matching was performed using the GNPS Library Search workflow, with the precursor ion mass tolerance set to 0.02 Da, fragment ion mass tolerance to 0.02 Da, a minimum cosine score threshold of 0.6, and requiring at least six matched fragment ions. Compound identification followed the Metabolomics Standards Initiative (MSI) guidelines (16) and was considered at Level 2 (putatively annotated compounds based on spectral similarity). The collision energies used in this study (20 eV and 40 eV) are consistent with those commonly applied for the acquisition of MS/MS spectra of aconitum alkaloids in the reference databases, thereby enhancing the reliability of spectral matching. Specifically, compounds were identified if they met the following stringent criteria: an accurate mass deviation of less than 5 ppm and an MS/MS spectral similarity score exceeding 60. All candidate matches were manually inspected to verify the quality of the MS/MS spectral alignments and minimize false positives. A simplified workflow from sample preparation through UHPLC-QTOF-MS analysis to GNPS-based identification was shown in Supplementary Figure S1.

## 3 Results

### 3.1 LC-QTOF-MS/MS analysis of herbal medicinal wine sample

**Figure 1:**
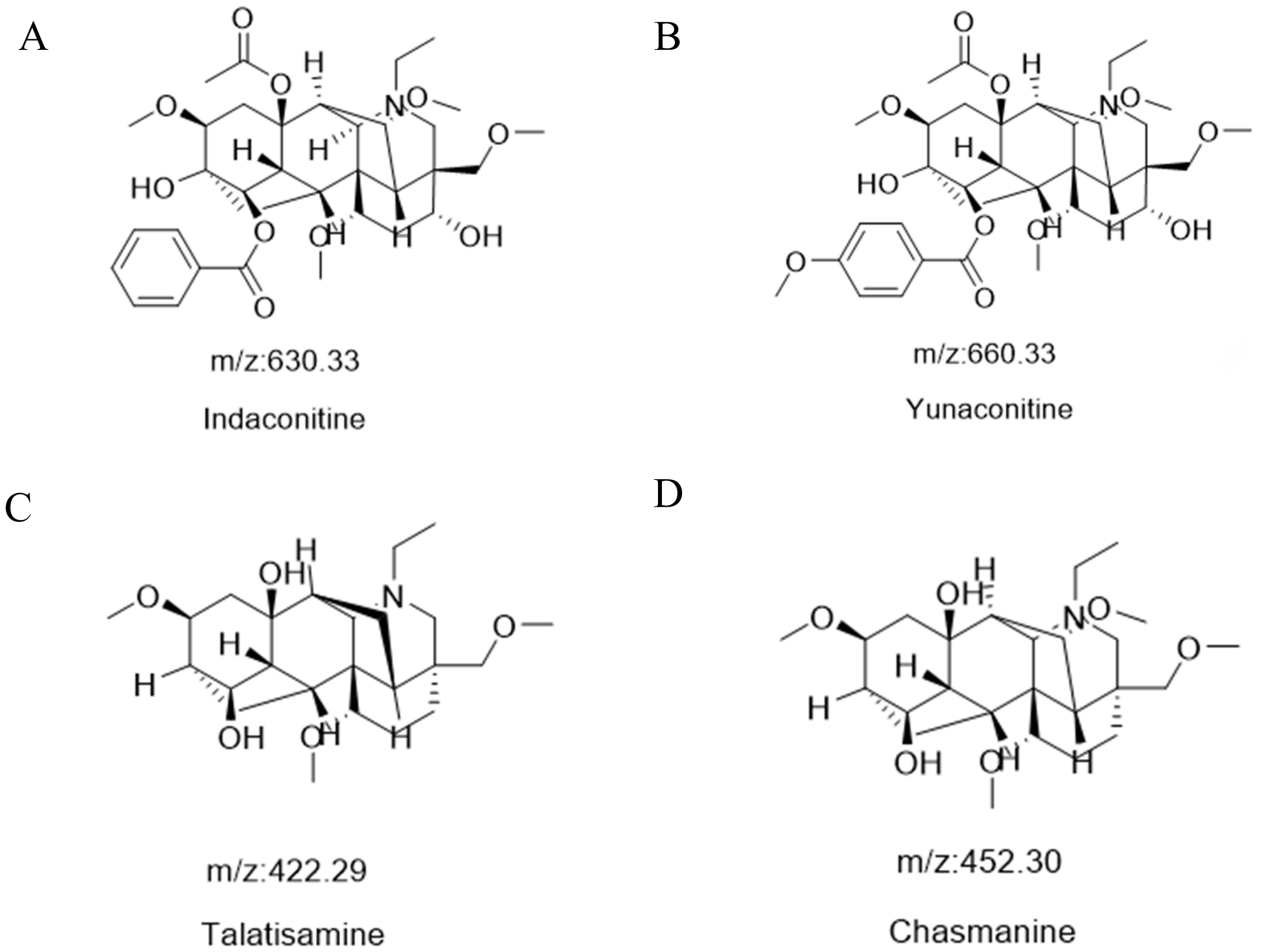
The two-dimensional chemical structure of indaconitine **(A)**, yunaconitine **(B)**, talatisamine **(C)** and chasmanine **(D)**.

**Figure 2:**
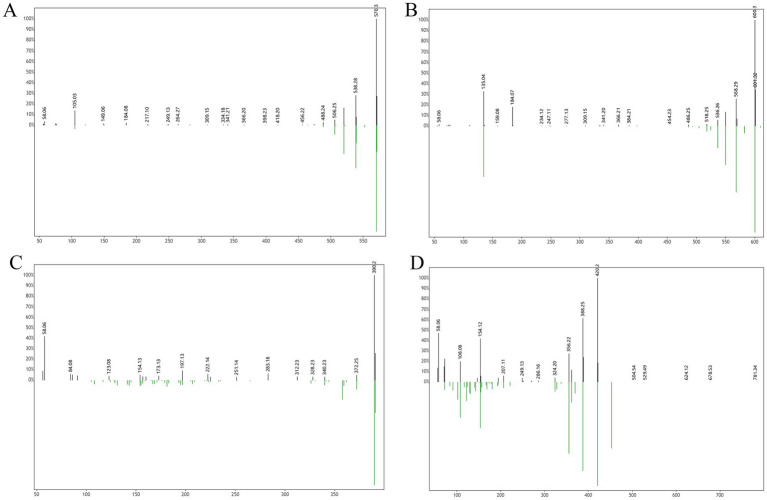
Mirror comparison of MS/MS spectra of indaconitine **(A)**, yunaconitine **(B)**, talatisamine **(C)** and chasmanine **(D)**. Top spectrum in each panel as the “Experimental Spectrum (from the herbal wine sample)” and the bottom spectrum as the “Reference Library Spectrum (from the GNPS/BMDMS-NP database).”

**Figure 3:**
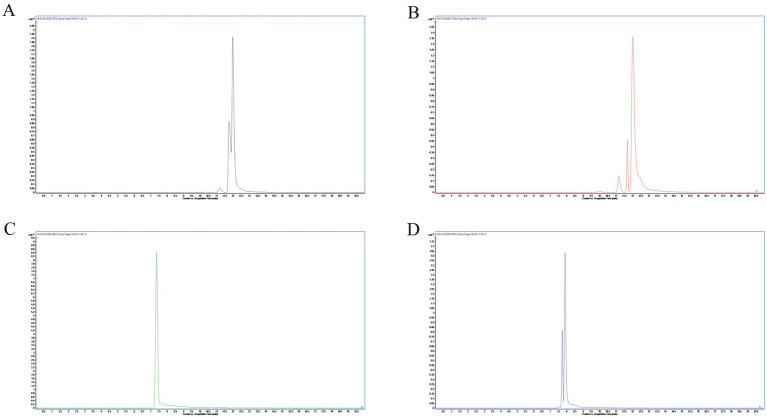
Extracted ion chromatograms (EICs) at ±10 ppm mass windows for [M + H]^+^ of indaconitine **(A)**, yunaconitine **(B)**, talatisamine **(C)** and chasmanine **(D)**.

**Figure 4:**
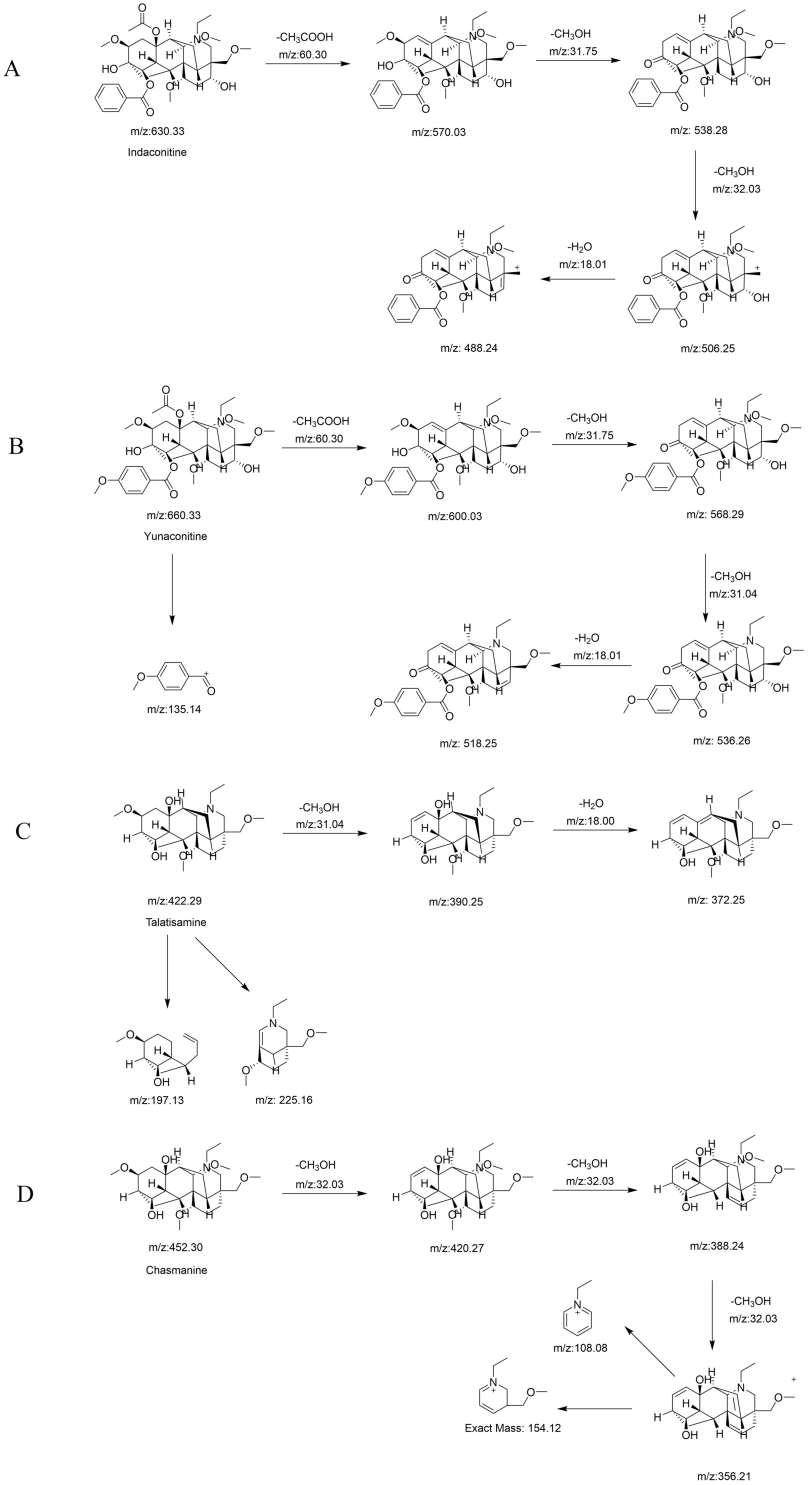
The possible fragmentation pathways and the formation of key product ions of indaconitine **(A)**, yunaconitine **(B)**, talatisamine **(C)** and chasmanine **(D)**.

Following a liquid–liquid extraction, 5 μL of the filtrate was injected into the UHPLC-HRMS system for analysis. Untargeted screening identified four alkaloids—indaconitine, yunaconitine, talatisamine, and chasmanine. The reference MS/MS spectra for indaconitine, yunaconitine, talatisamine, and chasmanine were sourced from the BMDMS-NP database, which contains high-quality reference data acquired under standardized conditions. The two-dimensional chemical structure of the four alkaloids was shown in Figure 1. Their theoretical mass, experimental mass, mass error, retention time and MS/MS spectral similarity score are summarized in Table 1. All detected compounds exhibited mass errors of less than 5 ppm. Specifically, indaconitine exhibited an accurate mass deviation of 0.95 ppm, and an MS/MS spectral similarity score of 93. Yunaconitine showed an accurate mass deviation of −2.42 ppm, and an MS/MS similarity score of 81. Talatisamine had an accurate mass deviation of 3.79 ppm, and an MS/MS similarity score of 64. Finally, chasmanine displayed an accurate mass deviation of −3.54 ppm, and an MS/MS similarity score of 73. Mirror comparison of MS/MS spectra of the four alkaloids were shown in Figure 2. Top spectrum in each panel as the “Experimental Spectrum (from the herbal wine sample)” and the bottom spectrum as the “Reference Library Spectrum (from the GNPS/BMDMS-NP database).” These results collectively supported the presence of these aconitum alkaloids in the medicinal liquor sample. Since none of these compounds were confirmed using reference standards, their identification was classified as MSI Level 2 (putatively annotated compounds based on spectral similarity). The typical extracted ion chromatogram of compounds is shown in Figure 3. The possible fragmentation pathways of the four alkaloids and the formation of key product ions are shown in Figure 4.

**Table 1:** Retention time, molecular formula, accurate mass of four alkaloids obtained by UHPLC-QTOF-MS.

Analyte	Formula	RT (min)	Protonated mass [M + H]^+^ (Da)		Mass error (ppm)	MS/MS similarity score	Characteristic fragment ions
			Theoretical	Experimental			
Indaconitine	C_34_H_47_NO_10_	10.7	630.3273	630.3279	0.95	93	570.03, 538.28, 506.25, 488.24
Yunaconitine	C_35_H_49_NO_11_	10.7	660.3378	660.3362	−2.42	81	600.03, 568.29, 536.26, 518.25, 135.04
Talatisamine	C_24_H_39_NO_5_	6.5	422.2901	422.2917	3.79	64	390.25, 372.25, 225.16, 197.13
Chasmanine	C_25_H_41_NO_6_	7.1	452.3007	452.2991	−3.54	73	420.2, 388.25, 356.22, 154.12, 108.08

### 3.2 Treatment and prognosis

A 54-year-old male patient with no significant prior medical history, comorbidities, or long-term medication use presented with acute symptoms following the ingestion of approximately 75 milliliters of a homemade medicinal liquor, consumed for the purpose of “kidney supplementation.” Within minutes of consumption, the patient began to experience abdominal discomfort and a burning sensation in the oral cavity. The symptoms progressively worsened, and approximately 1 h later, the patient developed profuse sweating, vomiting, and generalized weakness. He was subsequently brought to the emergency department by his family. The time elapsed from self-administration of the medication to hospital admission was approximately 1.5 h. Upon admission, the patient was fully conscious but exhibited signs of shortness of breath and intermittent vomiting. Blood pressure had escalated to 170/120 mmHg, and the heart rate exceeded 200 beats per minute, rapidly progressing to ventricular fibrillation. An urgent rescue operation was initiated, lasting for 6 h, during which 20–30 episodes of defibrillation were performed. Each episode of cardiac arrest did not exceed 2 min in duration. The patient was treated with continuous intravenous administration of lidocaine and amiodarone to manage arrhythmias, while oxygen therapy and suction were employed to prevent pulmonary infections. Oral activated charcoal was administered to facilitate the elimination of toxins, alongside gastric mucosal protection, fluid resuscitation, and blood glucose monitoring as part of a comprehensive therapeutic strategy. Following cardiopulmonary resuscitation (CPR), the patient exhibited the following laboratory values: lactate dehydrogenase (LDH) at 685.3 U/L, creatine kinase-MB (CK-MB) isoenzyme at 62.9 U/L, creatine kinase (CK) at 4889.3 U/L, troponin at 59.01 pg/mL. These findings indicate that the patient developed myocardial injury after CPR. Consequently, metoprolol extended-release tablets at a dosage of 47.5 mg were administered once daily to reduce myocardial oxygen consumption and control ventricular rate as part of the treatment regimen. After these interventions, the patient’s condition stabilized from a critical state, with stable vital signs and no significant discomfort. The patient was discharged in stable condition 2 weeks later.

## 4 Discussion

In China, *A. brachypodum* has been used as a traditional medicine and people still use *A. brachypodum* with or without a prescription. *A. brachypodum* contains various alkaloids, including bullatine A, bullatine B and aconitine alkaloids (3, 17). These diterpenoid alkaloids exhibit pharmacological activities such as analgesia, local anesthesia, anti-inflammation, and insecticidal effects. However, they also demonstrate inherent toxicity, primarily manifesting as cardiotoxicity and neurotoxicity. Due to factors such as geographic environment, the composition and content of *A. brachypodum* vary among different regions. All four alkaloids identified are diterpenoid aconitines, sharing similar mechanisms of neurotoxicity and cardiotoxicity. Among them, indaconitine and yunaconitine are particularly potent. It has been reported that intravenous administration of 0.05 mg/kg and oral administration of 2 mg/kg in mice induced symptoms such as restlessness, piloerection, and vomiting (18). These toxicological profiles highly align with the patient’s symptoms, including ventricular arrhythmia and vomiting, further supporting the role of these alkaloids as the causative agents of the poisoning.

Co-ingestion of aconitine and alcohol has been shown to significantly exacerbate the toxic effects of aconitine. Alcohol can rapidly enhance the absorption of aconitine in the bloodstream by promoting vasodilation and increasing the permeability of cellular membranes and increases the solubility of aconitine (19–21). These physiological changes result in faster systemic uptake, higher peak plasma concentrations, and a reduced time to toxicity onset, thereby lowering the threshold for lethal outcomes. Therefore, homemade medicinal wines containing aconitum roots exhibit significantly greater toxicity than traditional decoctions, making them an especially hazardous form of exposure. Further, aconite poisoning can produce symptoms similar to those of alcohol intoxication, such as dizziness, nausea, vomiting, and somnolence. Individuals poisoned by aconitine may mistakenly attribute these symptoms to being drunk, often missing the optimal window for treatment (22).

Given the lack of pathological features, identification of the herb responsible for the poisoning, and toxicological confirmation of aconitine or its metabolites remains crucial for the rescue of poisoned patients. Comprehensive toxicological screening particularly LC-MS/MS is indispensable. Analysis by LC/MS/MS may offer some advantages with respect to specificity as well as shorter preparation and analytical time. Compared to low-resolution mass spectrometry (LRMS), high-resolution mass spectrometry (HRMS), such as Q-TOF-MS and quadrupole-orbitrap, provide high-resolution mass spectrum under the full scan acquisition mode with better sensitivity and selectivity (excellent resolving power of up to 100,000 FWHM), and are therefore gaining more attention in poison identification (23, 24).

The application of large-scale public mass spectral databases such as GNPS offers considerable benefits for the identification of small molecules in toxic traditional Chinese medicines (TCMs). These databases house extensive, community-curated MS/MS spectral libraries that significantly improve the efficiency of identifying known toxic constituents in complex herbal matrices, eliminating the immediate need for purchasing or preparing reference standards for each compound. This approach is particularly valuable for toxic TCMs, which frequently contain diverse classes of hazardous compounds—such as alkaloids, glycosides, and other bioactive toxins—that pose serious health risks if improperly used. However, the reliability of spectral matching is fundamentally constrained by the coverage and quality of existing library spectra. Many toxic constituents unique to specific regional herbal practices or rare species may lack corresponding reference MS/MS data, resulting in lower confidence annotations or even complete oversight.

It is important to note that identifications based solely on spectral similarity are classified as MSI Level 2, meaning they are putative and lack confirmation with reference standards. This limitation carries significant clinical and toxicological implications, as misidentification could lead to inappropriate treatment or regulatory errors. False positives may arise from spectral overlaps or matrix effects, while false negatives may occur due to low abundance or absent library entries. Moreover, differences in sample preparation protocols, instrument platforms, and collision energy settings across studies may alter fragmentation patterns, potentially leading to mismatches or false identifications. Therefore, in follow-up studies, confirmation using authentic standards and orthogonal methods such as NMR or targeted tandem mass spectrometry is essential to validate these findings and support confident decision-making in clinical and regulatory contexts. Although the GNPS-based approach enabled the identification of several toxic aconitine alkaloids in this study, we were unable to confirm the presence of more specific markers such as bullatine A due to the lack of commercially available reference standards and the absence of its MS/MS spectrum in public databases at the time of this emergency analysis. As noted in previous studies (25, 26), bullatine A is a characteristic and abundant alkaloid in *A. brachypodum*. Its detection in future studies using validated reference materials would further substantiate the attribution of the poisoning source.

The integration of public mass spectral databases such as GNPS into standard toxicology workflows offers a complementary approach for rapid and broad-spectrum screening of toxic compounds, particularly in cases involving complex matrices like herbal preparations. Unlike targeted methods that require prior knowledge of suspected toxins, GNPS enables untargeted screening against a vast repository of community-curated spectra, facilitating the identification of both known and emerging toxins without the immediate need for reference standards (15). However, the adoption of such platforms in routine clinical settings faces several barriers, including the need for specialized training in mass spectrometry data interpretation, variability in instrument configurations affecting spectral reproducibility, and limitations in database coverage for region-specific or rare toxins. Despite these challenges, the continuous expansion of public databases and the development of user-friendly bioinformatics tools hold promise for broader implementation in clinical toxicology.

In summary, this study demonstrates the effectiveness of utilizing large-scale public mass spectral databases for the rapid identification of toxic small molecules in TCM preparations. By leveraging the extensive spectral libraries within GNPS, we were able to promptly identify several aconitum alkaloids—including indaconitine, yunaconitine, talatisamine, and chasmanine—directly from a suspected toxic TCM sample without the need for individual reference standards. This underscores the practical utility of such platforms in facilitating swift source attribution, supporting toxicological investigations, and aiding clinical decision-making in poisoning cases involving complex herbal formulations.

## Data Availability

The original contributions presented in the study are included in the article/Supplementary material, further inquiries can be directed to the corresponding authors.

## Footnotes

^1^ https://gnps.ucsd.edu

## Supplementary material

SUPPLEMENTARY FIGURE S1 The simplified workflow from sample preparation through UHPLC-QTOF-MS analysis to GNPS-based identification.
