# Supplementary figures and images for: When the cure turns toxic: a case report on toxic alkaloids identified by public mass spectral databases
Source: Front Med (Lausanne). 2025 Oct 20;12:1681334. doi: 10.3389/fmed.2025.1681334 (PMC12580361; doi:10.3389/fmed.2025.1681334)

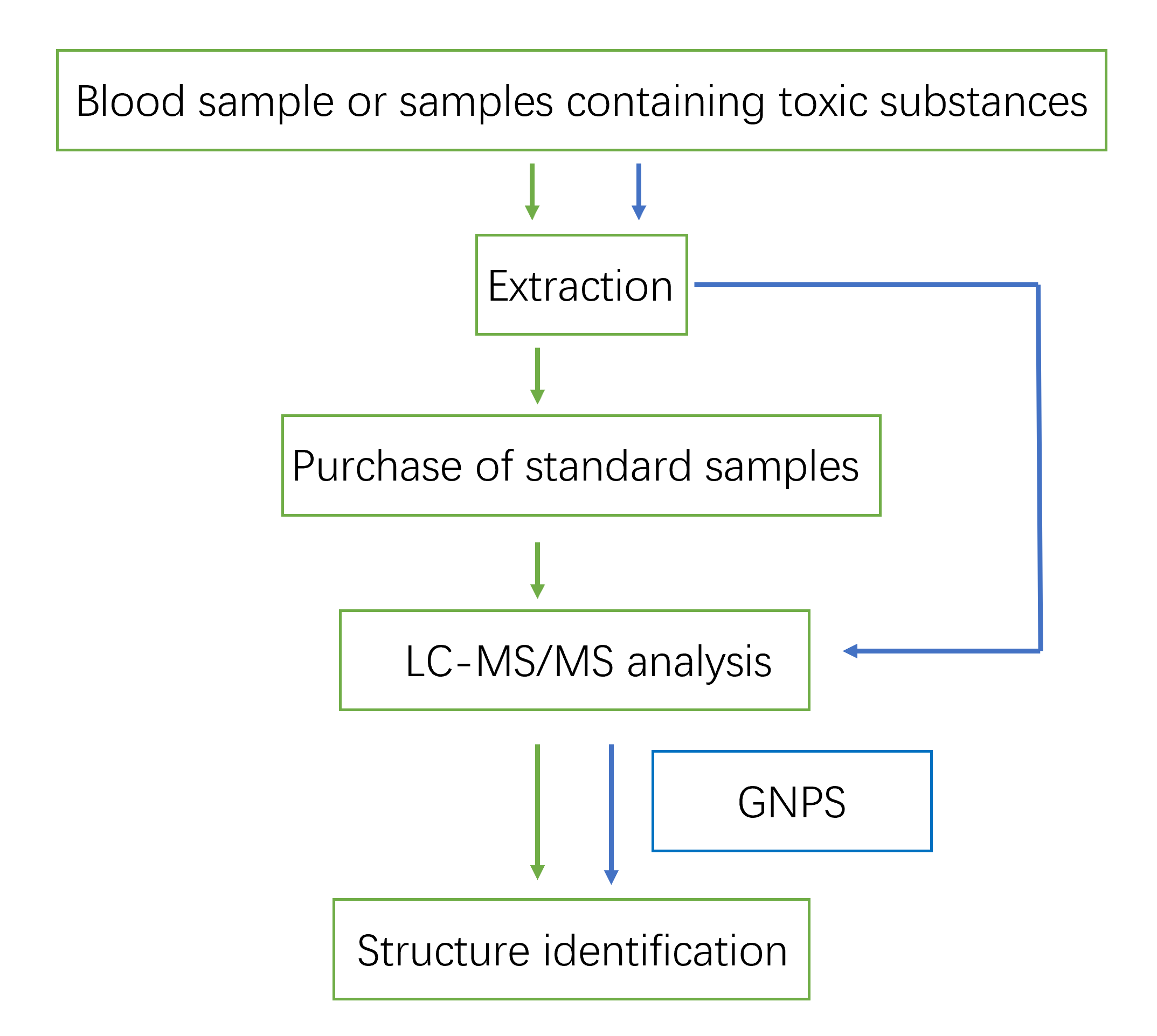

Supplement: SUPPLEMENTARY FIGURE S1 — The simplified workflow from sample preparation through UHPLC-QTOF-MS analysis to GNPS-based identification. [file Image_1.TIF]
